# Fused isoselenazolium salts suppress breast cancer cell growth by dramatic increase in pyruvate-dependent mitochondrial ROS production

**DOI:** 10.1038/s41598-020-78620-8

**Published:** 2020-12-09

**Authors:** Marina Makrecka-Kuka, Pavels Dimitrijevs, Ilona Domracheva, Kristaps Jaudzems, Maija Dambrova, Pavel Arsenyan

**Affiliations:** 1 Latvian Institute of Organic Synthesis, Aizkraukles 21, Riga, 1006 Latvia; 2 Riga Stradins University, Dzirciema 16, Riga, 1007 Latvia

**Keywords:** Cancer, Oncology

## Abstract

The development of targeted drugs for the treatment of cancer remains an unmet medical need. This study was designed to investigate the mechanism underlying breast cancer cell growth suppression caused by fused isoselenazolium salts. The ability to suppress the proliferation of malignant and normal cells in vitro as well as the effect on NAD homeostasis (NAD^+^, NADH, and NMN levels), NAMPT inhibition and mitochondrial functionality were studied. The interactions of positively charged isoselenazolium salts with the negatively charged mitochondrial membrane model were assessed. Depending on the molecular structure, fused isoselenazolium salts display nanomolar to high micromolar cytotoxicities against MCF-7 and 4T1 breast tumor cell lines. The studied compounds altered NMN, NAD^+^, and NADH levels and the NAD^+^/NADH ratio. Mitochondrial functionality experiments showed that fused isoselenazolium salts inhibit pyruvate-dependent respiration but do not directly affect complex I of the electron transfer system. Moreover, the tested compounds induce an immediate dramatic increase in the production of reactive oxygen species. In addition, the isoselenazolothiazolium derivative selectively binds to cardiolipin in a liposomal model. Isoselenazolium salts may be a promising platform for the development of potent drug candidates for anticancer therapy that impact mitochondrial pyruvate-dependent metabolism in breast cancer cells.

## Introduction

Breast cancer is the most common cancer in women and is the second leading cause of cancer-related deaths worldwide^1,2^. In clinical practice, a wide range of chemotherapeutic agents are used for treating cancer, but most of these agents are also toxic to nonmalignant cells and therefore cause serious side effects, which further worsen the patients’ well-being. Chemotherapeutic agents frequently lack selectivity, and their use may result in tumors becoming resistant to the drug^3^. Due to key role of mitochondria in cellular proliferation and death^4^, targeting mitochondria is a perspective anticancer strategy, that has reached clinical trial stage and received FDA approval. For example, venetoclax, an inhibitor of mitochondrial antiapoptotic protein Bcl-2, and enasidenib, an inhibitor of mitochondrial metabolic enzyme IDH2, were approved by the FDA for the treatment of chronic lymphocytic leukemia and acute myeloid leukemia, respectively^5^. Classic approaches targeting the mitochondria of cancer cells are usually aimed at directly affecting the functions of mitochondrial antiapoptotic proteins or inducing changes in energy metabolism (causing a shift from glycolysis to oxidative phosphorylation). Despite the well-known Warburg effect, cancer cells can also develop a hybrid metabolic phenotype (both glycolysis and oxidative phosphorylation to support ATP production), which ensures the plasticity of cancer cells in metastasis and therapy resistance^6^. Moreover, mitochondria participate in metabolic crosstalk with the tumor microenvironment, which plays a key role in the progression of breast cancer^7^. Thus, approaches targeting cancer mitochondrial bioenergetics (affecting metabolism and/or apoptotic pathways) are promising for the development of novel and effective anticancer therapeutics^8^.

Since selenium is involved in many signaling pathways in the human body and is vital for proper physiological performance and the prevention of cell mutations leading to oncological diseases^9^, the introduction of selenium into the structures of these molecules has attracted the attention of researchers^10,11^. Various research groups have focused on developing prospective drug candidates^11–20^; however, the most famous selenium drug candidate so far is ebselen, which contains Se–N bonds. It is a multifunctional compound that catalyzes several essential reactions (e.g., reduces reactive oxygen species (ROS) in a manner similar to glutathione peroxidase (GPx), and highly efficiently oxidizes reduced thioredoxin and catalyzes hydrogen peroxide reduction by thioredoxin reductase (TrxR), which acts as a dehydroascorbic acid (DHA) reductase mimetic) for the protection of cellular components from oxidative and free radical damage^21^. Recently, we elaborated methods for the preparation of novel, stable fused isoselenazolium salts, namely, systems with a Se–N^+^ bond^22^. These compounds, which possess electrophilic selenium in their structure, exhibit glutathione peroxidase-like properties, are toxic to *S. feltiae*, induce DNA double-strand damage at moderate doses, and display excellent antibacterial activity^23–25^. Interestingly, bacteria and mitochondria share a unique phospholipid in their membrane structures, cardiolipin (CL), which is a prospective therapeutic target^26–28^. CL was identified as a novel molecular signature of oncocytic and prostate tumors characterized by abnormally high abundance and chemical diversity^29–31^. Thus far, interactions of selenium-containing compounds with CL and the corresponding effects on mitochondrial function have not been studied.

In this report, we evaluated the ability of isoselenazolium salts to inhibit human breast adenocarcinoma MCF-7 and mouse carcinoma 4T1 proliferation. Their effects on rat cardiomyoblasts (H9C2), mouse fibroblasts (NIH 3T3), primary human epidermal keratinocytes (HEKa), Madin-Darby Canine Kidney (MDCK) cells, and rat vascular smooth muscle cells (A7r5) are included in this study to demonstrate whether these compounds are harmless to normal cells. The effects of these compounds on NAD^+^ homeostasis and mitochondrial function were studied. The ability of isoselenazolium salts to interact with CL-containing mitochondrial model membranes was evaluated by NMR spectroscopy and isothermal titration calorimetry (ITC).

## Results

### Fused isoselenazolium salts display up to nanomolar cytotoxic activity

**Figure 1:**
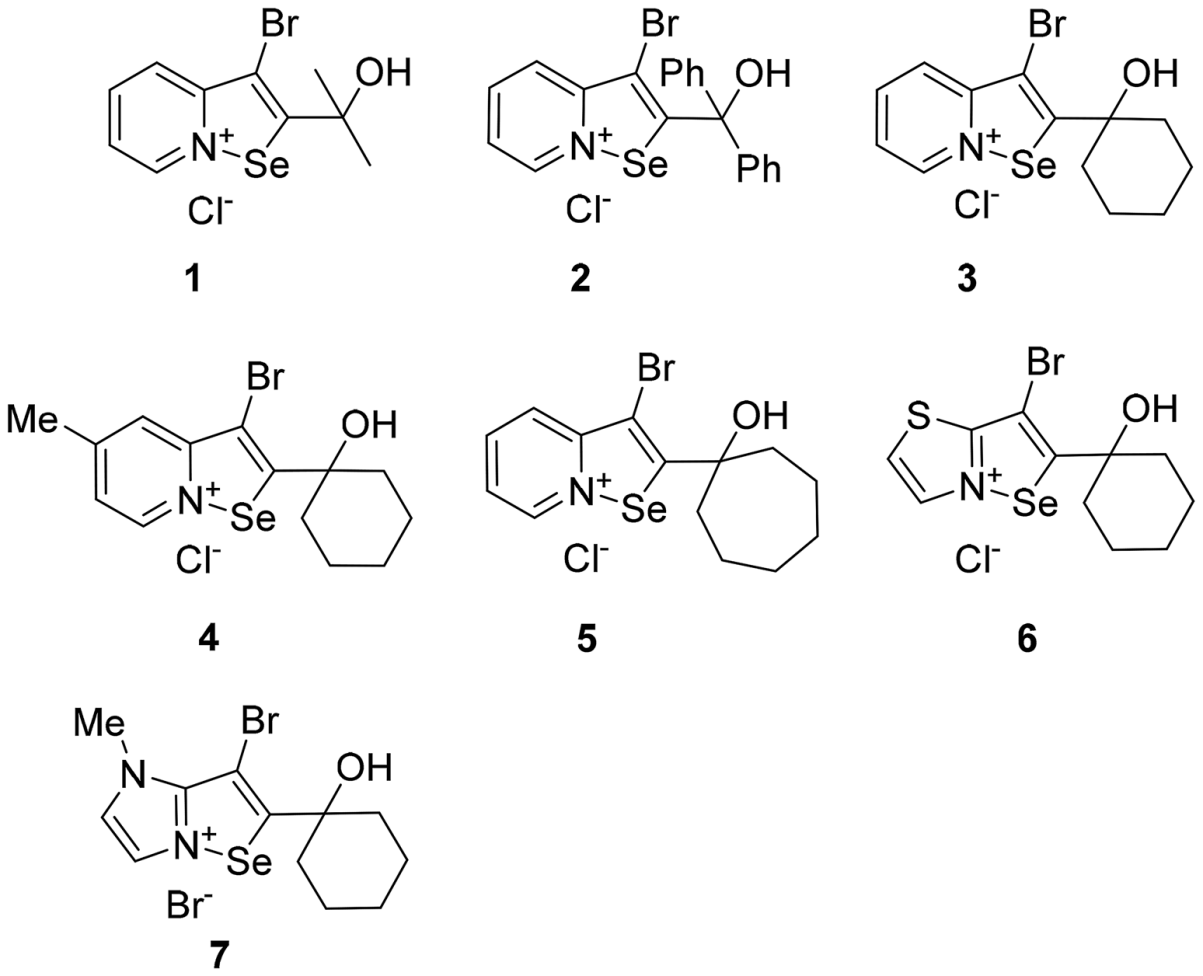
Tested fused isoselenazolium salts **1**–**7**.

The cytotoxicities of fused isoselenazolium salts **1**–**7** (Fig. 1) are summarized in Table 1. All the compounds demonstrated higher cytotoxic activities (IC_50_ values ranging from 0.044 to 3.23 µM) than the reference compound, Na_2_SeO_3_, in breast tumor cell lines. IC_50_ values of the studied isoselenazolium salts **1–7** were in the same range as for doxorubicin, however, mammary carcinoma (4T1) cells were more susceptible to derivative **3** than to doxorubicin. The IC_50_ values of compound **1** for the tumor cell lines were comparable to its IC_50_ values for normal cell lines. The replacement of the methyl group with a phenyl group (**2**) potentiated the cytotoxic effect on the MCF-7 breast tumor cell line. Notably, compound **3** showed the lowest cytotoxicity IC_50_ values (nanomolar range) against breast tumor cells. The introduction of a methyl substituent on the pyridine ring (**4**) led to a slight decrease in the cytotoxicity. Cyclohexyl ring (**4**) expansion to cycloheptyl (**5**) increased the cytotoxicity by approximately 3 times against MCF-7 cells. To study the influence of the heterocycle on the activity of the fused isoselenazolium salt, we replaced the pyridine with thiazole and imidazole rings. The introduction of a thiazole ring (**6**) decreased the cytotoxicity to tumor cells and to normal cell lines. However, [1,2]selenazolo[2,3-*b*]imidazolinium bromide (**7**) exhibited a slightly higher ability than compound **6** to suppress tumor cell growth. However, **7** showed higher toxicity than compound **6** against cardiomyocytes H9C2 and fibroblasts 3T3.

**Table 1:** Cytotoxic activity of **1**–**7** against breast tumor and normal cell lines.

Compound	Cytotoxicity, IC_50_, μM						
	Breast cancer cell lines		Normal cell lines				
	MCF-7	4T1	H9C2	3T3	HEKa	MDCK	A7r5
Na_2_SeO_3_	17.1 ± 2.4	4.9 ± 0.6	1.5 ± 0.3	22.3 ± 3.6	13.3 ± 1.1	6.3 ± 0.4	39.4 ± 8.2
Doxorubicin	1.0 ± 0.3	0.16 ± 0.06	11.0 ± 1.0	0.75 ± 0.09	n.t	57.0 ± 6.1	1.82 ± 0.35
**1**	3.10 ± 0.03	0.30 ± 0.03	1.8 ± 0.1	0.13 ± 0.02	2.31 ± 0.02	3.26 ± 0.40	2.89 ± 0.42
**2**	0.39 ± 0.03	1.1 ± 0.08	4.2 ± 0.3	1.6 ± 0.3	2.78 ± 0.08	6.27 ± 0.29	1.87 ± 0.26
**3**	0.50 ± 0.02	0.044 ± 0.005	2.2 ± 0.1	0.39 ± 0.05	2.21 ± 0.02	2.91 ± 0.13	1.85 ± 0.22
**4**	1.48 ± 0.04	0.45 ± 0.02	2.9 ± 0.1	0.79 ± 0.02	2.05 ± 0.05	2.54 ± 0.57	1.93 ± 0.16
**5**	0.29 ± 0.01	0.41 ± 0.05	9.3 ± 0.2	0.98 ± 0.03	2.01 ± 0.08	1.51 ± 0.37	1.92 ± 0.44
**6**	3.23 ± 0.03	1.7 ± 0.2	3.7 ± 0.2	7.4 ± 0.8	2.84 ± 0.08	6.58 ± 0.33	1.97 ± 0.27
**7**	1.48 ± 0.02	0.94 ± 0.06	0.67 ± 0.02	2.2 ± 0.3	2.19 ± 0.09	4.28 ± 0.34	1.66 ± 0.25

Values are shown as the means ± S.D. from 3 independent experiments.

nt, not tested.

In addition, IC_50_ values of **1–7** on HEKa and A7r5 were around 2 μM and did not depend on particular structure of isoselenazolium. Derivatives **2** and **6** were less toxic to MDCK cells than the other compounds. It is worth noting that 3-bromo-2-(1-hydroxycyclohexyl)-[1,2]selenazolo[2,3-*a*]pyridinium chloride (**3**) had the most pronounced selectivity towards tumor cell lines (> 42 fold, comparing 4T1 to A7r5). Derivatives **3**, **6** and **7** were chosen to study the possible mechanisms of action of fused isoselenazolium salts, taking into consideration the difference in cytotoxic effects on carcinoma and normal cell lines.

### Fused isoselenazolium salts affect on NAD^+^ homeostasis

**Figure 2:**
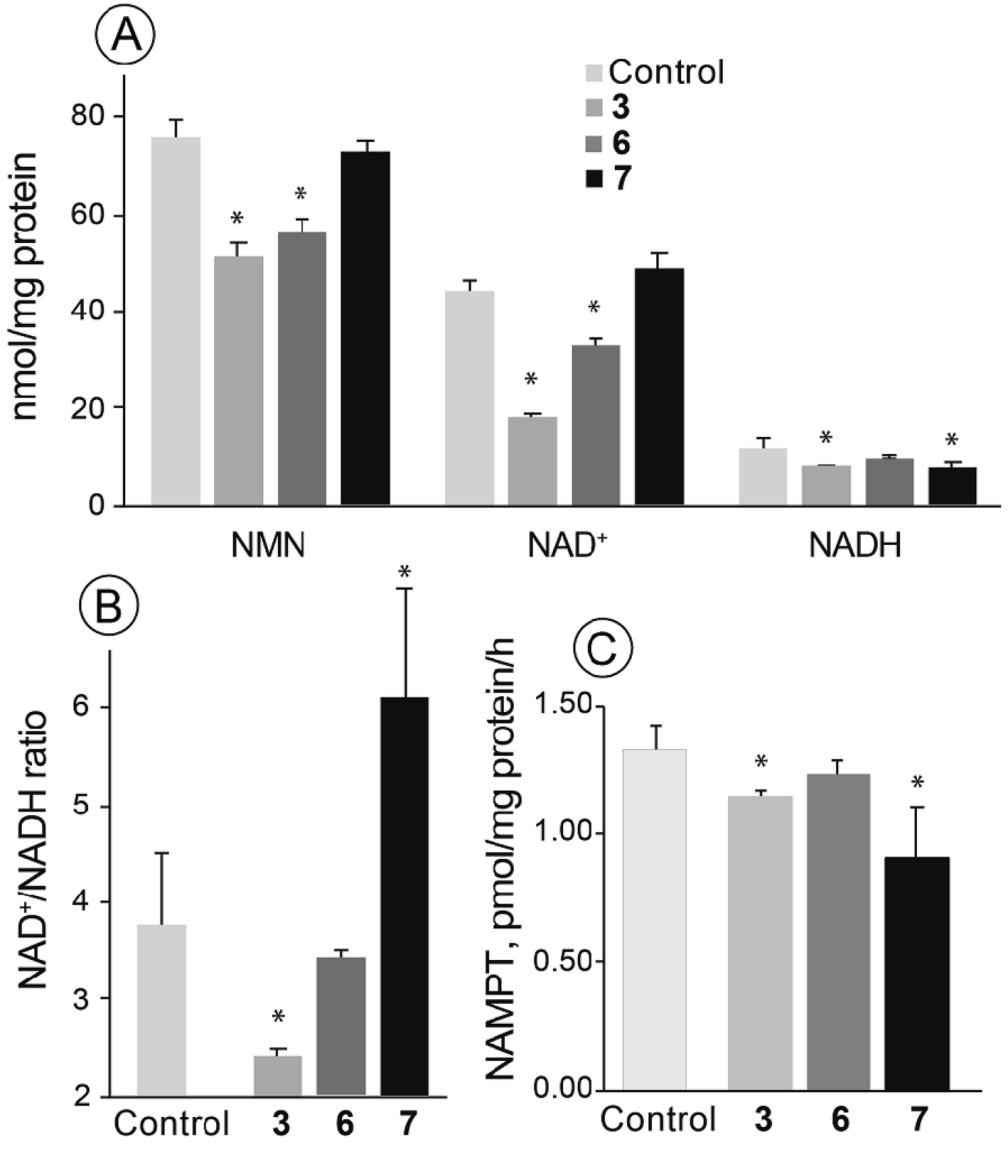
The effects of fused isoselenazolium salts on the levels of NMN, NAD^+^ and NADH and NAMPT activity in MCF-7 cells (**A**); NAD^+^/NADH ratio (**B**); NAMPT inhibition by **3**, **6** and **7**. Values are shown as the mean ± S.D. (*n* = 6). Significant difference (*- *p* < 0.05) compared with control.

**Figure 3:**
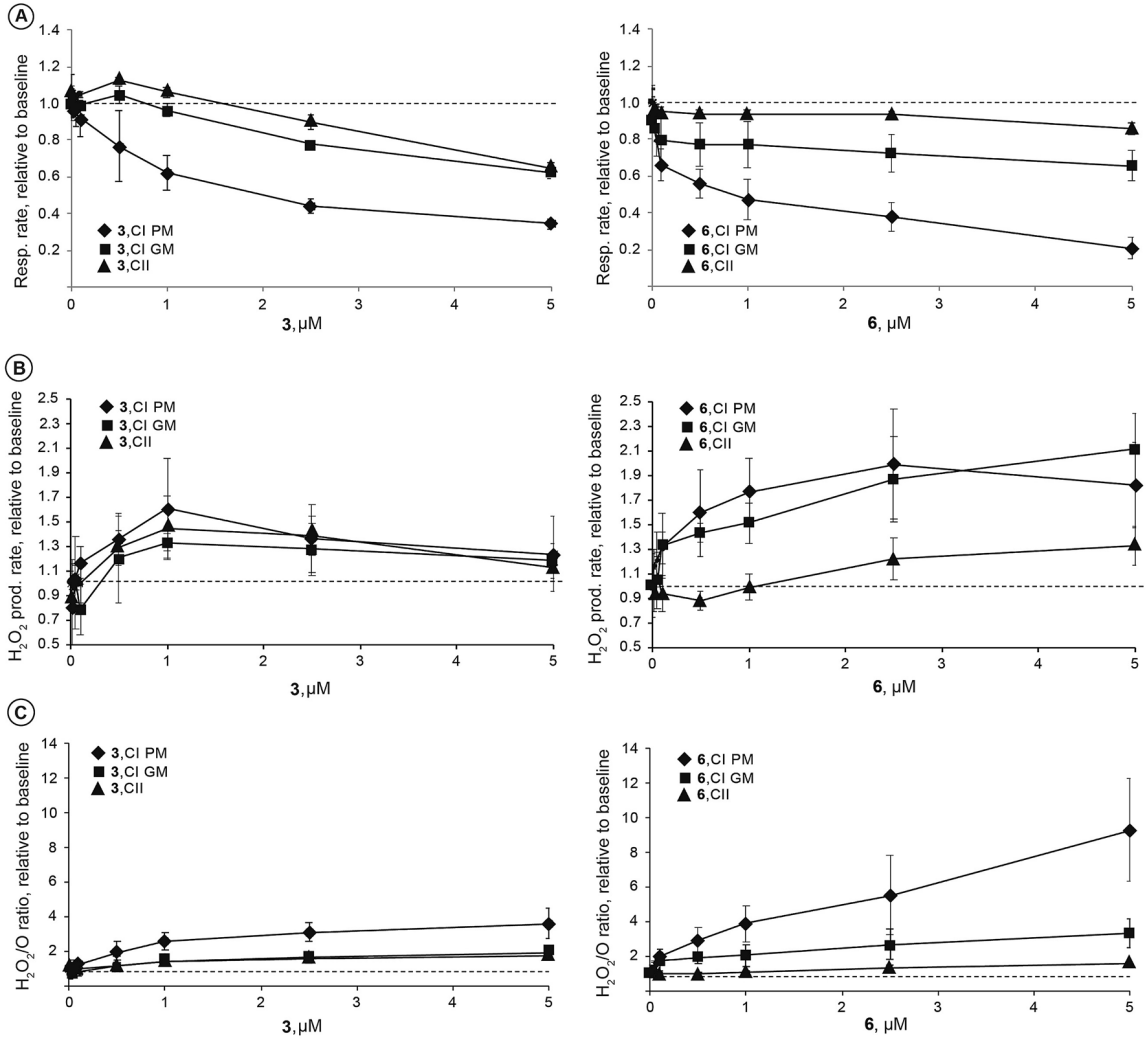
The concentration-dependent effects of compounds **3** and **6** on mitochondrial function in permeabilized 4T1 cells. Concentration dependent changes in mitochondrial respiration rate (**A**) and H_2_O_2_ production rate (**B**) and H_2_O_2_/O ratio (**C**) at Complex I or II linked OXPHOS. P—pyruvate; M—malate; G—glutamate. Values are shown as mean ± S.D. (n = 3–5 experiments) relative to baseline (dashed line)—before addition of the compound.

The levels of NAD^+^ and its metabolites are critical for tumor cell proliferation^32–35^; therefore, the effects of the fused isoselenazolium salts on the NMN, NAD^+^ and NADH levels as well as NAMPT activity were tested. As shown in Fig. 2A, compounds **3** and **6**, but not compound **7**, induced a decrease in NMN levels by 33% and 26%, respectively. In addition, compound **3** significantly decreased the levels of NAD^+^ and NADH by 59% and 33%, respectively, and as a result, the NAD^+^/NADH ratio was decreased by 38% (Fig. 2A,B). Compound **6** induced a 25% decrease in the NAD^+^ level without affecting the NADH content or the NAD^+^/NADH ratio in the cells (Fig. 3A,B). In contrast to compound **6**, compound **7** induced a decrease in NADH level without affecting the NAD^+^ concentration, and as a result, the NAD^+^/NADH ratio was 1.65-times higher than that in untreated cells (Fig. 2A,B). Notably, only isoselenazoloimidazolium salt **7** induced a notable reduction in NAMPT activity (by 31%, Fig. 2C). Taken together, these results indicate that fused isoselenazolium salts alter NAD^+^ homeostasis in breast tumor cells.

### Fused isoselenazolium salts inhibit pyruvate-dependent mitochondrial respiration and facilitate ROS production

Since mitochondria play a significant role in ROS production and NAD^+^ metabolism and experiments showed that fused isoselenazolium salts affect NAD^+^ homeostasis, the next step was to test whether the compounds alter mitochondrial function. First, the concentration-dependent effects of compounds **3** and **6** on the complex I (NADH-linked) and complex II (succinate-linked) pathways were determined. Both compounds, **3** and **6**, inhibited the mitochondrial respiration rate in a concentration-dependent manner and increased H_2_O_2_ production with complex I (CI, NADH-linked) substrates (both pyruvate + malate and glutamate + malate) (Fig. 3A,B). In addition, there were no significant changes in mitochondrial function in the CII-linked OXPHOS state (Fig. 3). Despite difference in compound potency, the most pronounced increase in the H_2_O_2_/O ratio in the presence of isoselenazolium salts **3** and **6** was observed when pyruvate and malate were used as substrates (Fig. 3C). These results indicate that isoselenazolium salts most likely affects pyruvate-dependent mitochondrial metabolism.

**Figure 4:**
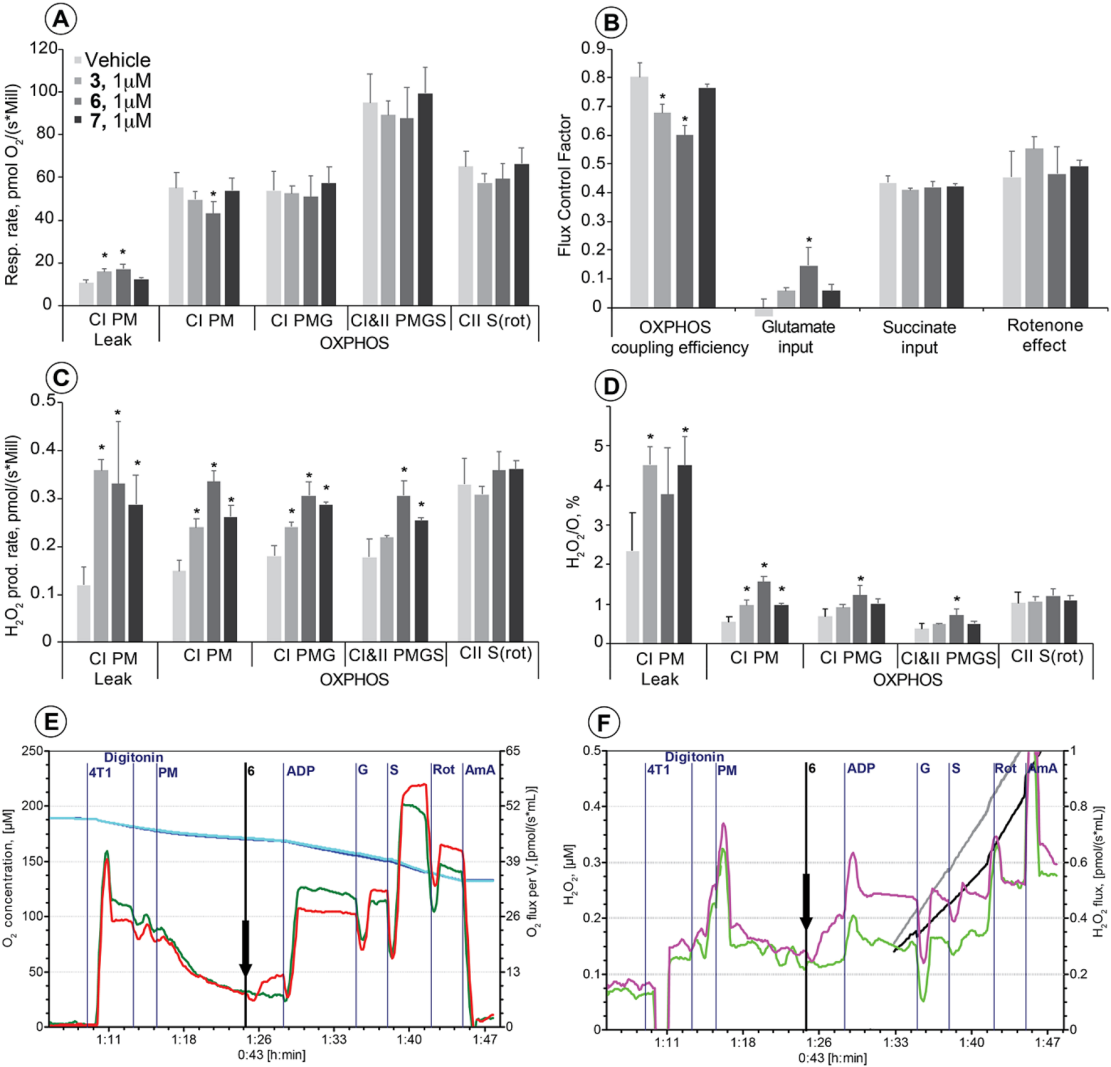
The effect of fused isoselenazolium salts at 1 μM concentration on mitochondrial respiration (**A**), flux control factors (**B**), H_2_O_2_ production rate (**C**) and H_2_O_2_/O ratio (**D**) in permeabilized 4T1 cells. Representative traces of respiration (**E**) and H_2_O_2_ production rate (**F**) measurement (vehicle—green (**E**) and light green (**F**) lines; compound 6—red (**E**) and purple (**F**) lines). CI—complex, I; CII—complex II; LEAK—substrate dependent respiration rate; OXPHOS—oxidative phosphorylation dependent state; P—pyruvate; M—malate; ADP—saturating ADP; G—glutamate; S—succinate; Rot—rotenone, AmA—antimycin A. OXPHOS coupling efficiency corresponds to 1-Respiratory Control Ratio^-1^. Flux Control Factor indicates on the input of each substrate and/or pathway to the electron transfer system performance. Values are shown as mean ± S.D. (n = 3–4 experiments). Significant difference (*- *p* < 0.05) compared with control.

**Figure 5:**
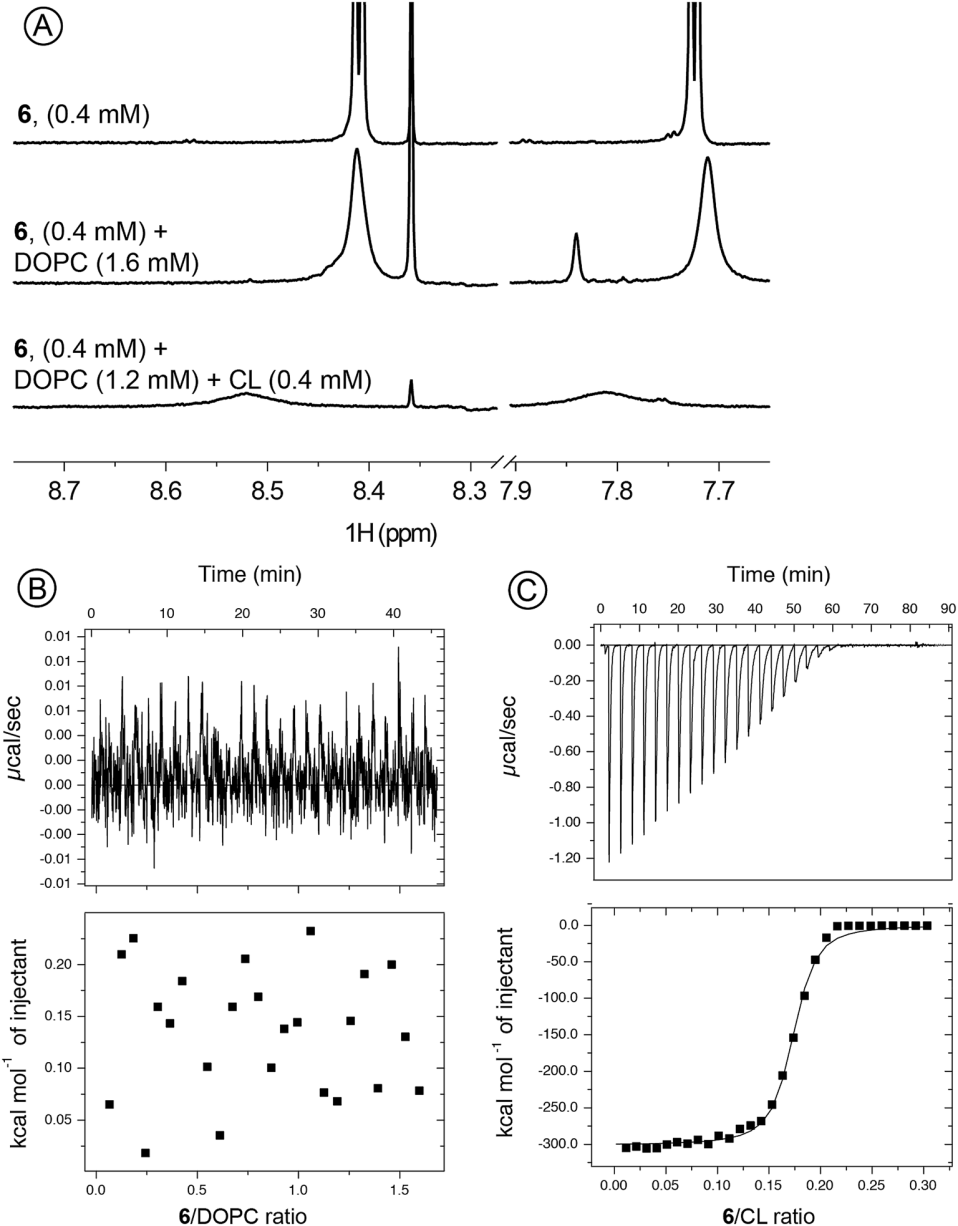
(**A**) 600 MHz ^1^H NMR spectra of 0.4 mM **6** (top), 0.4 mM **6** and 1.6 mM DOPC (middle) and 0.4 mM **6** and 1.2 mM DOPC with 0.4 mM CL (bottom) in 90% H_2_O/10% D_2_O. (The zoomed aromatic region is shown. Full spectra presented in SI); (**B**) and (**C**) Binding isotherms and calorimetric curves at 25 °C for titration of DOPC and CL/DOPC (1:3) containing vesicles with serial injections of compound **6**.

To determine whether the observed effects of the fused isoselenazolium salt are related to the inhibition of the pyruvate-dependent pathway but not to the direct inhibition of complex I, the mitochondrial function in permeabilized breast cancer cells was evaluated in the presence of 1 µM **3, 6** and **7**. Although only compound **6** decreased the respiration rate in the OXPHOS state with pyruvate and malate (Fig. 4A,E), both compounds **3** and **6** induced a significant decrease in the OXPHOS coupling efficiency (corresponds to 1-Respiratory Control Ratio^-1^) (Fig. 4B; ESI Fig S1). The addition of another NADH-dependent complex I substrate, glutamate, compensated for the decrease in respiration with pyruvate + malate, as shown by the increased flux control factor (characterizes individual substrate/pathway input to the electron transfer system performance) for glutamate (Fig. 5B). Together with the unchanged flux control factor for rotenone (Fig. 4B), these results indicate that fused isoselenazolium salts do not inhibit complex I. All three tested isoselenazolium derivatives (**3, 6** and **7**) induced immediate significant increases in H_2_O_2_ production and the H_2_O_2_/O ratio in the pyruvate-dependent OXPHOS state (Fig. 4C,D,F). The effects of compound **6** on H_2_O_2_ production and the H_2_O_2_/O ratio were the most pronounced (2.3- and 2.8-fold increases, respectively). In addition, significant increases in H_2_O_2_ production and the H_2_O_2_/O ratio in the CI&II-linked OXPHOS state were observed in cells treated with **6** (Fig. 4C,D). Taken together, the obtained results indicate that fused isoselenazolium salts inhibit pyruvate-dependent mitochondrial respiration and facilitate ROS production.

### Fused isoselenazolium salts selectively interact with cardiolipin

To evaluate whether fused isoselenazolium salts can act directly on mitochondria, the interactions of derivative **6** with mitochondrial membrane components, DOPC/CL liposomes, were tested. As seen in Fig. 5A, in the absence of DOPC and CL, compound **6** shows sharp resonances with clearly distinguishable spin coupling patterns. In the presence of DOPC liposomes, the aromatic resonances of compound **6** are noticeably broadened such that the coupling pattern is not resolved. This could indicate a weak interaction between compound **6** and DOPC liposomes or, alternatively, the changes in resonance could be due to altered molecular surroundings (liposomal dispersion). However, in the presence of CL-containing liposomes, these resonances are broadened. These results were also confirmed by the ITC experiments (Fig. 5B,C). DOPC liposome titration with compound **6** did not result in any heat release, and no signs of binding were observed (Fig. 5B). In contrast, the negative heat flow observed after each injection of compound **6** (in μcal/s) indicates that the isoselenazolium cation–CL interaction is accompanied by a decrease in enthalpy (Fig. 5C). These results indicate that compound **6** binds to the mitochondrial model membrane.

## Discussion

The discovery of a mechanism of action is a corner stone in the development of new drug candidates for anticancer therapy. In the study presented in herein, we sought to “lift the veil” and determine the nature of cancer cell growth suppression by isoselenazolium salts.

**Figure 6:**
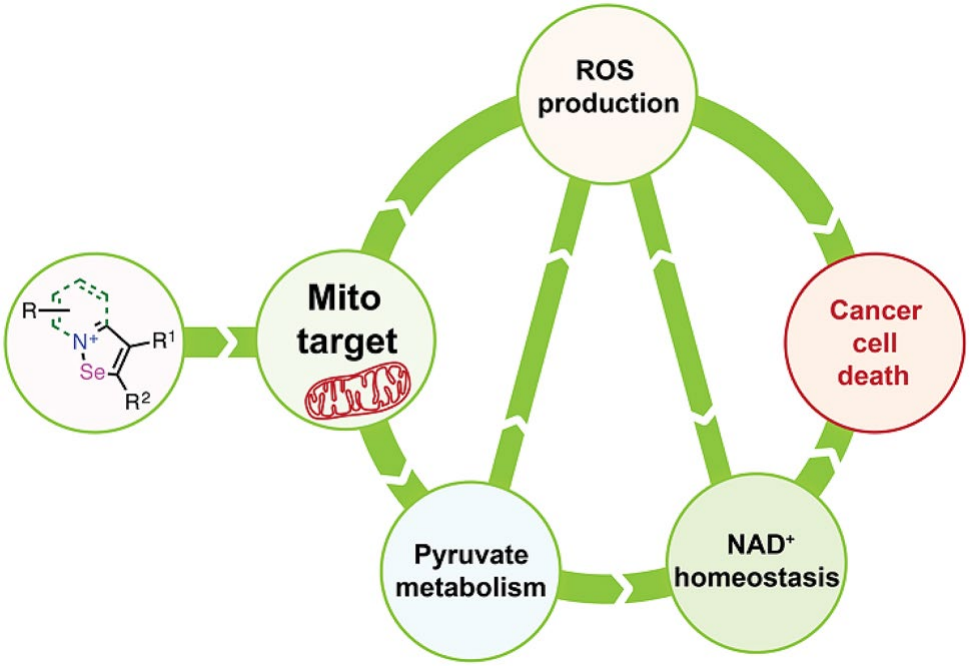
Proposed cytotoxicity-inducing mechanism of action of isoselenazolium salts.

Here, we show for the first time that isoselenazolium salts exhibit in vitro cytotoxic effects against breast cancer cells at nanomolar to low micromolar concentrations. These compounds are more cytotoxic on studied cancer cell lines than widely known doxorubicin (up to 3.6 fold on 4T1 cell line), moreover, some derivatives are equally or less harmful to normal cells. The IC_50_ values of doxorubicin in tumor cells versus cardiomyocytes are 10–100-fold lower, alongside some of studied isoselenazolium salts showed similar tendency. Thus, we can expect that therapeutic potential of presented compounds could be comparable to doxorubicin, however, to maximize it, improvement of isoselenazolium salts selectivity towards tumor cells is required. Furthermore, our study shows the potential mechanisms responsible for the anticancer activities of fused isoselenazolium salts. The compounds inhibit pyruvate metabolism and simultaneously increase ROS production, resulting in altered NAD^+^ homeostasis in breast cancer cells. Based on differences in cytotoxic effects against malignant and normal cells, isoselenazolium salts could be a powerful platform for further optimization to develop agents that target cancer cell mitochondrial energy metabolism. The studied derivatives significantly increased mitochondrial ROS production and inhibited pyruvate-dependent metabolism (Fig. 6), thus showing cancer cell-specific cytotoxic activity.

Targeting mitochondrial metabolic pathways and the redox homeostasis of tumor cells is an attractive approach for anticancer therapy^36–38^. Isoselenazolium salts have intriguing effects on the main metabolic pathways in tumor cells and are associated with cell bioenergetics, affecting mitochondrial NAD^+^/NADH-dependent metabolism. The NAD^+^/NADH ratio plays an important role in the regulation of the intracellular redox state and proliferation, impaired cell death signaling, and deregulated metabolism^32,39^. The induction of excess NAD^+^ over NADH is an attractive approach for the prevention of tumor metastasis in vivo^33^. Although NAMPT inhibition has been proposed as an effective strategy for disturbing NAD^+^ homeostasis in cancer cells^34^, only slight decreases in NAMPT activity were observed with **3** and **7**, and this result could not explain the changes in NAD^+^ metabolite levels induced by isoselenazolium salts. Thus, it is more likely that fused isoselenazolium salts alter NAD^+^ homeostasis by acting on NAD^+^-dependent pathways (e.g. SIRT and PARP family enzymes, Preiss-Handler pathway) or on the energy metabolism processes, rather than on NAD^+^ biosynthesis. The mechanisms of action could be partly related to the inhibition of pyruvate metabolism, as breast cancer cell proliferation relies heavily on pyruvate metabolism^40^. The inhibition of pyruvate metabolism by a blockade of the mitochondrial pyruvate transporter was recently shown to decrease the uptake of extracellular lactate, thus inhibiting lactate-driven metabolic symbiosis between cancer cells^41^. These metabolic changes resulted in sensitization of cancer cells to oxidative stress and inhibition of cell growth. Isoselenazolium salts block pyruvate-dependent mitochondrial respiration without affecting complex I, leading to the selective disruption of cancer cell metabolism and dramatically increased ROS production. Thus, isoselenazolium salts exhibit dual activity: they both inhibit pyruvate-dependent metabolism and simultaneously induce oxidative stress that results in the inhibition of cell division and causes cytotoxic effects.

The isoselenazolium salt-stimulated pyruvate metabolism-driven ROS production could be explained by several mechanisms^42^: (a) effect on electron transfer system; (b) effect on tricarboxylic acid cycle (TCA); (c) effect on pyruvate metabolism pathway. The main sites of ROS production in electron transfer system are complexes I and III^43^. Since there is no change in ROS production in the presence of rotenone, i.e. in the conditions when complex I is inhibited, we can conclude that isoselenazolium salts do not affect complex III. Another option is that isoselenazolium salt-induced ROS production could be linked to the reverse electron transport via complex I. However, titration experiments with compound 6 at complex I-linked oxidative phosphorylation-dependent state showed that there is a significant difference in ROS production between complex I substrates used (pyruvate or glutamate), indicating that it is more likely that isoselenazolium salt-stimulated ROS production is not related to the induction of reverse electron transport process. Another source of increased pyruvate-driven ROS production could be 2-oxoglutarate dehydrogenase in TCA cycle^44^. The substrate flux through 2-oxoglutarate dehydrogenase is supported by both pyruvate and glutamate metabolism, thus, in the case of inhibition of 2-oxoglutarate dehydrogenase isoselenazolium salts would affect metabolism with both substrates. Our data showed that isoselenazolium salts do not inhibit glutamate metabolism; moreover, we observed the stimulation of glutamate metabolism in the presence of pyruvate after addition of tested compounds, as indicated by increased flux control factor (ESI, Fig. S1). Thus, the involvement of 2-oxoglutarate dehydrogenase in the isoselenazolium salt-induced ROS production could be excluded. Other proposed mechanism of action is the inhibition of pyruvate metabolism by either inhibiting pyruvate transport or pyruvate dehydrogenase complex (PDC). Since compounds at tested concentrations did not affect the pyruvate dehydrogenase activity (ESI Fig. S2), it is more likely that isoselenazolium salts alter pyruvate transport; however, the involvement of PDC inhibition cannot be fully excluded.

Since the mitochondrial membrane potential (∆ψ_m_) in cancer cells is at least 60 mV higher than that of normal cell mitochondria^45^, positively charged molecules could be a useful approach for selectively targeting cancer cell mitochondria. Moreover, the accumulation of lipophilic cations inside the membrane is dependent on the membrane potential and increases by ten-fold for every 61.5 mV^45–47^. NMR and ITC experiments confirmed that fused isoselenazolium salt **6** selectively interacts with CL, a component of the inner mitochondrial membrane. Obviously, such selective binding takes place between a positively charged isoselenazolium core and negatively charged CL phosphate moieties. The ability of the isoselenazolium salt to bind to CL could explain the observed inhibition of pyruvate-dependent metabolism and the induction of ROS production, particularly because a similar mechanism of action was described for doxorubicin, an anthracycline antibiotic widely used as an anticancer agent^48^. However, unlike doxorubicin, at the studied concentrations, isoselenazolium salts do not inhibit electron transfer but specifically affect pyruvate-dependent metabolism, indicating they may have less pronounced cardiotoxic side effects. Taking into account doxorubicin-induced cardiotoxicity, further studies will be focused on the cardiac safety of the presented isoselenazolium compounds for the further development of novel isoselenazolium derivatives with improved cytotoxic selectivity to cancer cells compared with normal cells, particularly cardiomyocytes.

## Methods

### Tested compounds

Isoselenazolium salts were synthesized, purified and characterized by spectroscopy and elemental analysis as described before^22^:

3-bromo-2-(2-hydroxy-2-methylethyl)-[1,2]selenazolo[2,3-*a*]pyridinium chloride (**1**).

3-bromo-2-(hydroxydiphenylmethyl)-[1,2]selenazolo[2,3-*a*]pyridin-8-ium (**2**).

3-bromo-2-(1-hydroxycyclohexyl)-[1,2]selenazolo[2,3-*a*]pyridinium chloride (**3**).

3-bromo-2-(1-hydroxycyclohexyl)-5-methyl-[1,2]selenazolo[2,3-*a*]pyridin-8-ium (**4**).

3-Bromo-2-(1-hydroxycycloheptyl)-[1,2]selenazolo[2,3-*a*]pyridinium bromide (**5**).

3-bromo-2-(1-hydroxycyclohexyl)[1,2]selenazolo[2,3-*b*]thiazolium chloride (**6**).

3-bromo-*N*-methyl-2-(1-hydroxycyclohexyl)[1,2]selenazolo[2,3-*b*]imidazolium bromide (**7**).

Sodium selenite, Na_2_SeO_3_, a simple and toxic selenium compound, was used as a reference.

### Cell culture

MCF-7 (human breast adenocarcinoma, estrogen-positive) and 4T1 (mouse carcinoma), as breast cancer cell lines, and H9C2 (rat cardiomyoblasts), NIH 3T3 (mouse fibroblasts), HEKa (primary human epidermal keratinocytes), MDCK (Madin-Darby Canine Kidney) cells, and A7r5 (rat vascular smooth muscle cells), as normal cell lines, were obtained from the American Type Culture Collection [ATCC] collection for use in the current study. All cell lines were cultured in Dulbecco's modified Eagle's medium (DMEM) containing 10% fetal bovine serum (FBS) and 4 mM L-glutamine at 37 °C and 5% CO_2_.

### Cytotoxicity assay^49^

Cell viability was assessed by the addition of 3-(4,5-dimethylthiazol-2-yl)-2,5-diphenyltetrazolinium bromide (MTT). Briefly, cells were seeded (2–6 × 10^4^ cells/ml) in 96-well plates and allowed to attach for 24 h. Solutions of test compounds were prepared and serially diluted to obtain the appropriate concentrations. The cells were treated with the test compounds at different concentrations (0.032–100 µM) and incubated for 48 h at 37 °C and 5% CO_2_. Then, the culture medium was removed, and medium containing 0.2 mg/ml MTT was added. After 3 h (37 °C, 5% CO_2_), the MTT-containing medium was removed, and 200 μl of dimethyl sulfoxide (DMSO) was immediately added to each sample. The absorbance was assessed at 540 nm using a Tecan multiplate reader Infinite 1000 (Austria). The half-maximal inhibitory concentration (IC_50_) of each compound was calculated using Graph Pad Prism 3.0.

### Measurement of NMN, NAD^+^ and NADH levels

For fluorometric measurements of NMN, a previously described method was used, with minor modifications^50^. Briefly, MCF-7 cells (1 × 10^5^ cells per well) were seeded in a 24-well plate. After 24 h, cells were treated with 3 selected compounds (2 μM) for 10 min. The cells were washed with PBS and then lysed by adding 200 μl of 1 M HClO_4_ to each well and incubating at 4 °C for 20 min. The cells were harvested by scraping and transferred to a tube. The extracts were neutralized with 100 μl of 2 M KOH for 5 min at room temperature and 50 μl of 0.1 M bicine (pH 7.4) was added. The samples were centrifuged at 14 000 × g for 10 min at 4 °C, and the supernatant was collected for further analysis. The protein concentration in each sample was determined using a Bio-Rad protein assay kit. Twenty-five microliters of sample or standard solution (0.3 to 20 μM NMN) was transferred into a flat-bottom 96-well black plate and mixed with 10 μl of 2 M KOH and 10 μl of ice-cold 20% acetophenone. The reaction was incubated for 2 min at 4 °C, after which 45 μl of 88% formic acid was added, and the samples were incubated for another 10 min at 37 °C on a shaker. The fluorescence was measured using a Tecan multiplate reader Infinite 1000 (Austria) with excitation at 382 nm and emission at 420 nm.

The NAD^+^/NADH ratio in the MCF-7 cells exposed to the test compounds (2 μM) for 10 min was measured using an NAD^+^/NADH assay kit (Abcam, ab65348) according to the manufacturer's instructions.

### NAMPT activity assay

MCF-7 cells (5 × 10^6^ cells per well) were grown in a 6-well plate and then incubated with the test compounds (2 µM) for 4 h. The cells were harvested by scraping, washed with PBS and lysed with 50 mM Tris–HCl (pH 7.5), 150 mM NaCl, 1 mM DTT, 1 mM PMSF, 0.002 mg/ml leupeptin and pepstatin (2 × 10^6^ cells in 100 µl buffer solution). After three freeze–thaw cycles at -80 °C, the cell extract was centrifuged at 15 000 × g for 30 min at 4 °C. The supernatant was collected, and the protein concentrations were determined using a Bio-Rad protein assay kit. The NAMPT enzyme reaction was performed as described previously^51^. The enzymatic reaction mixture contained 50 mM Tris–HCl buffer (pH 7.5), 12 mM MgCl_2_, 2 mM DTT, 0.02% BSA, 2.5 mM ATP, 0.8 mM PRPP, and 50 μM nicotinamide. To initiate the reaction, 25 μl of cell extract was added to the reaction mixture, and the samples were incubated for 1.5 h at 37 °C. The enzymatic reaction was stopped by incubation at 95 °C for 1 min, and then samples were cooled and centrifuged at 10 000 × g for 5 min. The concentrations of the NAMPT reaction product, nicotinamide mononucleotide (NMN), in the samples were determined using a described method^52^. Twenty microliters of each reaction mixture was transferred to a 96-well black plate, and then 10 μl of 2 M KOH and 10 μl of 20% acetophenone in DMSO were added to the wells. After incubation on ice for 2 min, 45 μl of 88% formic acid was added. The fluorescence was measured using a Tecan multiplate reader Infinite 1000 (Austria) with excitation at 382 nm and emission at 445 nm. The NMN concentrations in the reaction mixtures were determined by comparison with NMN standards in the range of 0.16–20 μM. The NAMPT activity in the cell lysates is expressed as the concentration of NMN in μmol per mg protein per hour based on standard NMN readings.

### High-resolution fluorespirometry^53^

High-resolution fluorespirometry was performed using an Oxygraph-2 k system (OROBOROS INSTRUMENTS, Austria). All experiments were performed at 37 °C in MiR05 medium (110 mM sucrose, 60 mM K-lactobionate, 0.5 mM EGTA, 3 mM MgCl_2_, 20 mM taurine, 10 mM KH_2_PO_4_, 20 mM HEPES, pH 7.1 at 30 °C, and 0.1% BSA essentially fatty acid free). The medium was reoxygenated when the oxygen concentration dropped to 80 µM. H_2_O_2_ flux was simultaneously measured with respirometry in the O2k-Fluorometer using the H_2_O_2_-sensitive probe Ampliflu Red (AmR) as described^53^. The H_2_O_2_/O flux ratio [%] was calculated as the H_2_O_2_ flux/(0.5 O_2_ flux).

In permeabilized 4T1 cells, pyruvate + malate (5 and 2 mM) or glutamate + malate (10 and 2 mM) were used to determine complex I (CI)-linked LEAK (L) respiration. ADP was added to a concentration of 5 mM to determine the oxidative phosphorylation-dependent respiration (OXPHOS state). Succinate (10 mM, complex II (CII) substrate) was then added to reconstitute convergent CI&II-linked respiration. Rotenone (0.5 µM, an inhibitor of complex I) and antimycin A (2.5 µM, an inhibitor of complex III) were added to determine the CII-linked OXPHOS capacity and residual oxygen consumption (ROX), respectively. Compound **6** was added to permeabilized cells after the addition of the respective substrates in the OXPHOS state (titration experiment) or in the LEAK state. In addition, compounds **3**, **6** and **7** were tested at 1 µM in the OXPHOS coupling protocol in the LEAK state. The OXPHOS coupling efficiency was calculated as:

$$1 - \frac{{Re sp.rate\;LEAK\;state}}{{Re sp.rate\;OXPHOS\;state}}$$

The substrate-dependent flux control factor was calculated as:

$$1 - \frac{{{{Re}} sp.rate\;before\;addition\;of\;respective\;substrate}}{{{{Re}} sp.rate\;after\;addition\;of\;respective\;substrate}}$$

### Nuclear magnetic resonance studies

Cardiolipin (CL) liposomes for NMR analysis were prepared by the thin-film method followed by sonication and extrusion. The desired amount of CL stock solution (5 mg/ml, in ethanol, Avanti Polar Lipids, Inc.) and 1,2-dioleoyl-sn-glycero-3-phosphocholine (DOPC) stock solution (25 mg/ml, CHCl_3_, Avanti Polar Lipids) were added to a round-bottom flask, and the organic solvents were removed using a rotary evaporator. The obtained lipid thin film was dried *in vacuo* overnight. Then, the dry lipid film was rehydrated in D18 HEPES buffer (20 mM, pH 7.4, 10% D_2_O) and gently vortexed, and the samples were sonicated for 30 min in a bath-type sonicator. The obtained sample of liposomes was extruded 21 times through a 100-nm pore polycarbonate filter (Nucleopore Corp., CA). Similarly, liposomes from DOPC only were prepared.

To study the interactions of compound **6** with CL and DOPC liposomes, a stock solution of **6** (2 mM) in D18 HEPES buffer (20 mM, pH 7.4, 10% D_2_O) was added to CL/DOPC or DOPC liposome dispersions to reach 400:1200:400 µM CL/DOPC/**6** solution or 1600:400 µM DOPC/**6** solution followed by gentle vortexing for 30 s.

NMR spectra were acquired on a 600‐MHz Bruker Avance NEO spectrometer equipped with a 5 mm QCI-F quadruple resonance pulsed‐field‐gradient cryoprobe. The temperature was calibrated against methanol and set to 25 °C. Chemical shifts were referenced to the water resonance at 4.77 ppm with respect to 4,4-dimethyl-4-silapentane-1-sulfonic acid (DSS). The samples contained 0.8 mM **6** in 90% H_2_O and 10% D_2_O with and without DOPC or CL/DOPC liposomes. The NMR data were processed and analyzed using MestReNova software.

### Isothermal titration calorimetry studies^54,55^

Liposomes for isothermal titration calorimetry (ITC) studies were prepared by the thin-film method: the desired volume of DOPC (25 mg/ml, CHCl_3_, Avanti Polar Lipids) and CL (5 mg/ml, EtOH, Avanti Polar Lipids) stock solutions were concentrated to dryness under reduced pressure, and the lipid films were resuspended in HEPES buffer (20 mM, pH 7.4) to prepare 50:150 µM CL/DOPC or 200 µM DOPC liposome dispersions. The obtained large multilamellar liposomes were sonicated in a bath-type sonicator (Cole Parmer Ultrasonic Cleaner 8891CPX (USA)) at room temperature for 30 min and then extruded (LiposoFast-Basic, Avestin) through a 100 nm polycarbonate filter 21 times. The quality of the resulting small unilamellar vesicles was determined by dynamic light scattering (DLS) (Zetasizer Nano ZSP, Malvern Panalytical Ltd., UK).

The experiments were carried out using an isothermal titration calorimeter (MicroCal iTC200, Malvern Panalytical Ltd., UK). To carry out the titration, the injection syringe was filled with a solution of compound **6** (100 µM), and the reaction cell was filled with liposome dispersion (200 µM of total lipid). The experiments were performed at 25 and 37 °C with a stirring speed of 750 rpm. The titration was conducted with thirty 1-μl injections with 180 s intervals between injections to ensure complete equilibration. The data consisted of a series of heat flows as a function of time, which were collected automatically and analyzed by Origin 7 software (OriginLab Corporation, Northampton, USA, www.originlab.com, 2002). The changes in enthalpy (ΔH) due to the interactions between **6** and the liposome dispersions were recorded, and then stoichiometry (N), the association constant (K_A_), the dissociation constant (K_D_), entropy (ΔS) and the Gibbs free energy (ΔG) were calculated. The obtained sigmoidal titration curves were evaluated assuming independent saturable binding sites in the outer vesicle interface. The integration of the enthalpograms was carried out using the one-site binding model (1:1).

### Statistical analysis

All in vitro experiments were repeated at least three times. Data are presented as the mean ± standard deviation (S.D.). Statistically significant differences in the mean values were evaluated using one-way ANOVA. If ANOVA provided *p* < 0.05, Dunnett’s test was performed. The differences were considered significant when *p* < 0.05. The data were analyzed using Graph Pad Prism software (Graph Pad Inc., La Jolla, USA).

## Conclusions

Isoselenazolium salts with electrophilic selenium are a promising heterocyclic system for further elaboration as antitumor drug candidates. Our data confirm that isoselenazolium salts bind to the mitochondrial membrane-specific lipid CL, indicating the possible direct mitochondria-targeting ability of these salts. Furthermore, these compounds suppress cancer cell growth by modulating tumor cell energy metabolism and inducing marked ROS production. Our future studies will be focused on the development of novel derivatives containing isoselenazolium moieties with improved selective uptake by cancer cells compared with normal cells, especially cardiomyocytes.

## Supplementary information

Supplementary Information.
