# Supplementary material for: Fused isoselenazolium salts suppress breast cancer cell growth by dramatic increase in pyruvate-dependent mitochondrial ROS production
Source: Sci Rep. 2020 Dec 9;10:21595. doi: 10.1038/s41598-020-78620-8 (PMC7725824; doi:10.1038/s41598-020-78620-8)
Supplement: Supplementary file 1 — Supplementary Information. [file 41598_2020_78620_MOESM1_ESM.pdf]

Supplementary materials

Fused isoselenazolium salts suppress breast cancer cell growth by dramatic increase in pyruvate-dependent mitochondrial ROS production

M. Makrecka-Kuka,[a] P. Dimitrijevs,[a,b] I. Domracheva,[a] K. Jaudzems,[a] M. Dambrova,[a,b] P. Arsenyan [a]*

[a] *Latvian Institute of Organic Synthesis, Aizkraukles 21, LV-1006, Riga, Latvia. e-mail: pavel@osi.lv*

[b] *Riga Stradins University, Dzirciema 16, Riga, LV-1007, Latvia*

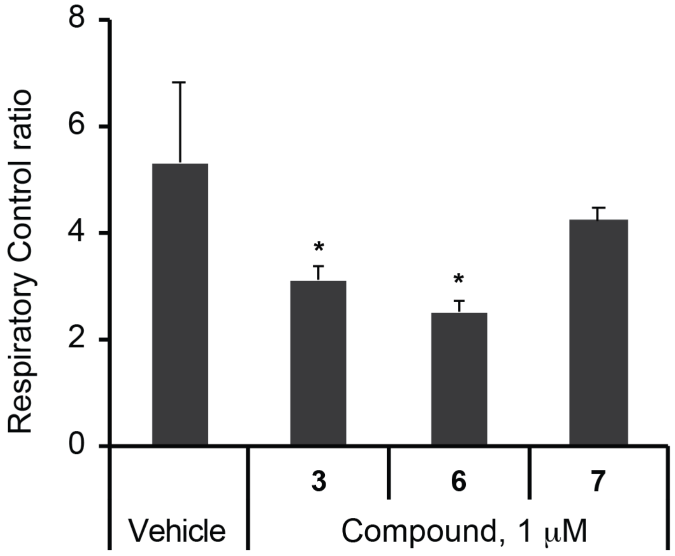

Figure S1. The effect of fused isoselenazolium salts at 1 μM on Respiratory Control Ratio (RCR). Values are shown as mean ± S.D. (n=3-4 experiments). Significant difference (*- $p<0.05$) when compared with control.

Respiratory Control Ratio is calculated as OXPHOS/LEAK state respiration ratio.

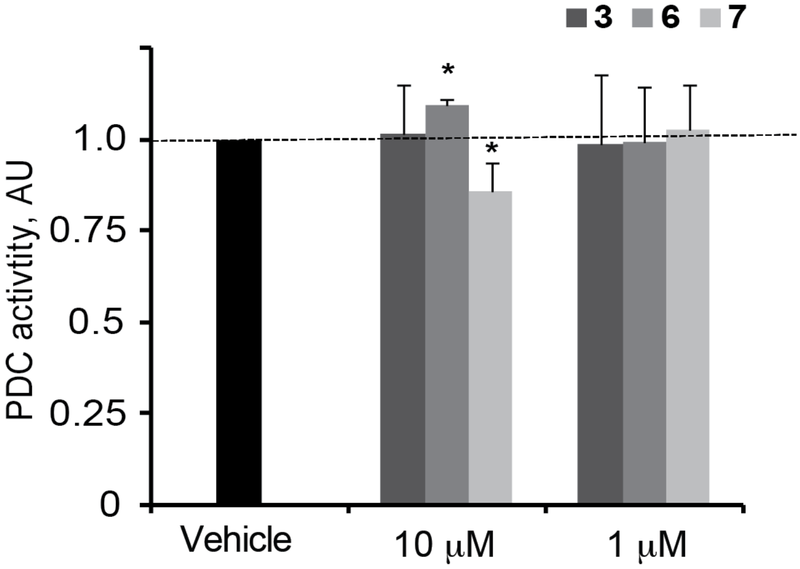

Figure S2. Pyruvate dehydrogenase complex (PDC) activity in the presence of 10 or 1 μM fused isoselenazolium salts. Values are shown as mean ± S.D. (n=4-5 independent measurements). Significant difference (*- $p<0.05$) compared with vehicle.

The pyruvate dehydrogenase complex (PDC) activity was measured as described previously[1] with slight modifications. Briefly, PDC from porcine heart (75 mU/mL, containing intrinsic pyruvate dehydrogenase kinases, obtained from Sigma-Aldrich Cat No. P7032) was incubated with tested compounds or vehicle at 37 °C for 40 min in reaction buffer containing 40 mM MOPS, 0.5 mM EDTA, 30 mM KCl, 1.5 mM $MgCl_2$, 0.25 mM acetyl-CoA, 0.05 mM NADH, 2 mM dithiothreitol, 10 mM NaF. Then, reaction buffer supplemented with 55 μM ADP and 100 μM ATP was added to the enzyme to trigger the kinase reaction. After 3 min, the kinase reaction was terminated by the addition of 55 mM ADP and pyruvate. At last, the remaining PDC activity was tested in at 37 °C by addition of 90 μL of buffer containing 120 mM Tris, 0.61 mM EDTA, 0.73 mM $MgCl_2$, 2.2 mM thiamine pyrophosphate, 11 mM 2-mercaptoethanol, 2.2 mM NAD+, 2.2 mM pyruvate, 1.1 mM CoA and then were measured the formation of NADH at 340 nm for 5 min. The measurement of PDC activity with vehicle in the absence of inhibitor was taken as 100%.

Reference

1. Wu, D.-M., Wang, Y.-J., Fan, S.-H., Zhang, Z.-F., Shan, Q., Lu, J., Chen, G.-Q., Zheng, Y.-L. High-throughput screening of novel pyruvate dehydrogenase kinases inhibitors and biological evaluation of their in vitro and in vivo antiproliferative activity, *Eur. J. Med. Chem.* **164**, 252–262 (2019).
